# Extracellular matrix bioink boosts stemness and facilitates transplantation of intestinal organoids as a biosafe Matrigel alternative

**DOI:** 10.1002/btm2.10327

**Published:** 2022-04-26

**Authors:** Zi‐Yan Xu, Jin‐Jian Huang, Ye Liu, Can‐Wen Chen, Gui‐Wen Qu, Ge‐Fei Wang, Yun Zhao, Xiu‐Wen Wu, Jian‐An Ren

**Affiliations:** ^1^ Research Institute of General Surgery, Affiliated Jinling Hospital, Medical School of Nanjing University Nanjing Jiangsu Province China; ^2^ School of Medicine, Southeast University Nanjing Jiangsu Province China; ^3^ Department of General Surgery, BenQ Medical Center Nanjing Jiangsu Province China

**Keywords:** bioprinting, dECM‐ink, intestinal organoids, photo‐crosslinking, stemness, transplantation

## Abstract

Organoids hold inestimable therapeutic potential in regenerative medicine and are increasingly serving as an in vitro research platform. Still, their expanding applications are critically restricted by the canonical culture matrix and system. Synthesis of a suitable bioink of bioactivity, biosecurity, tunable stiffness, and printability to replace conventional matrices and fabricate customized culture systems remains challenging. Here, we envisaged a novel bioink formulation based on decellularized extracellular matrix (dECM) from porcine small intestinal submucosa for organoids bioprinting, which provides intestinal stem cells (ISCs) with niche‐specific ECM content and biomimetic microstructure. Intestinal organoids cultured in the fabricated bioink exhibited robust generation as well as a distinct differentiation pattern and transcriptomic signature. This bioink established a new co‐culture system able to study interaction between epithelial homeostasis and submucosal cells and promote organoids maturation after transplantation into the mesentery of immune‐deficient NODSCID‐gamma (NSG) mice. In summary, the development of such photo‐responsive bioink has the potential to replace tumor‐derived Matrigel and facilitate the application of organoids in translational medicine and disease modeling.

## 1 INTRODUCTION

The intestinal epithelium hosts short‐lived differentiated cells of diverse lineages with a renewal cycle of 4–5 days and immortalized proliferative ISCs, which reside at the bottom of crypts.^1^ This subtle homeostasis of the epithelium depends on bidirectional gradients of proliferative and differentiated signals established by interspersed Paneth cells and intestinal stromal components, including intestinal subepithelial myofibroblast (ISEMFs), macrophages and endothelial cells, which are involved in the maintenance of ISCs via intracellular pathways such as Wnt/R‐spondin, BMP and Notch signaling.^2^, ^3^, ^4^, ^5^

**FIGURE 1:**
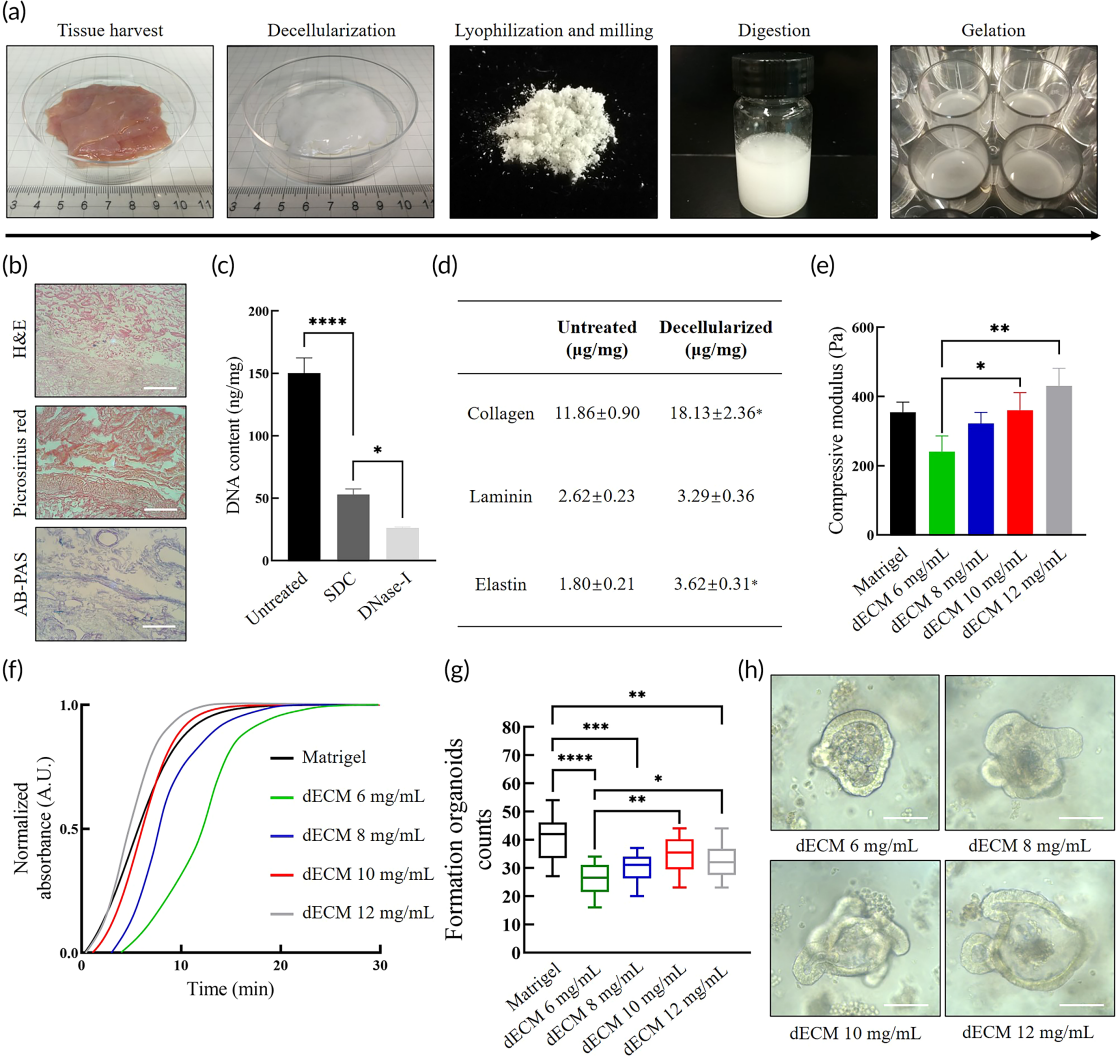
Characterization of decellularized extracellular matrix (dECM) hydrogel. (a) The preparation and gelation processes of dECM powder including harvesting of small intestine from freshly killed piglets, decellularization of the submucosa, lyophilization and milling into powder, sterilization, digestion in pepsin and HCl, adjustment of pH and salinity and incubation at 37°C. (b) Qualitative analysis of decellularized small intestine tissue by histological section including hematoxylin and eosin (H&E) for nucleus, Picrosirius Red (PR) for collagens and Alcian blue‐periodic acid‐Schiff (AB‐PAS) for glycosaminoglycans (GAGs). Scale bar 100 μm. (c) Quantitative analysis of DNA content in fresh untreated intestinal tissue, sodium deoxycholate (SDC)‐treated submucosa, and DNase‐treated submucosa. Mean ± SD (*n* = 3 batches). One‐way ANOVA. *p < 0.05 and ****p < 0.0001. (d) Quantitative ELISA analysis of dECM pregel including collagen, laminin, and elastin. Mean ± SD (*n* = 3 batches). Two‐sided *t*‐test *p < 0.05. (e) Compressive modulus measured by compressive testing. Mean ± S.D. (*n* = 3 samples). One‐way ANOVA. *p < 0.05 and **p < 0.01. (f) Turbidity analysis of dECM gels and Matrigel by spectrophotometry during heat‐mediated gelation. (g) Formed intestinal organoids per field of view at 100× at Day 7 at first passage. Mean ± S.D. (*n* = 16 from four organoids cultures). One‐way ANOVA. *p < 0.05, **p < 0.01, ***p < 0.001, and ****p < 0.0001. (h) Typical bright field images of formed organoids at Day 5 at first passage within dECM hydrogels. Scale bar 100 μm

The identification of leucine‐rich repeat‐containing G‐protein coupled receptor 5 (Lgr5) as a marker of ISCs enabled isolation and in vitro expansion of ISCs.^6^ Later, Sato et al.^7^ found that ISCs exhibited a regular expansion and differentiation pattern and formed self‐assembly 3D aggregates within Matrigel, an extract from Engelbreth‐Holm‐Swarm sarcoma with low mechanical properties (354.50 ± 29.37 Pa) (Figure 1e).^8^ Such ISC‐based micron‐sized 3D multicellular constructs with projecting crypt‐like buds and sealed‐off lumen are named intestinal organoids.

Despite the potentials in regenerative medicine and disease modeling, conventional organoid culture patterns have limitations.^9^, ^10^ First, encapsuled organoids within the hydrogel dome lack maneuverability and homogeneity. Second, canonical organoid culture pattern hardly reaches designed deposition of ISCs and refined tissue models for therapeutic application.^11^ Third, spatially variant biochemical distribution caused by dome‐like constructs can result in different maturation and differentiation levels of cultured organoids.^12^ Some of these drawbacks are attributed to Matrigel.^13^ As an exclusive ECM analog for organoid culture, Matrigel has increasingly imposed constraints on organoid applications. Potential contamination resulting from tumor origin leads to uncertain biosecurity and tumorigenicity. Component variations among batches cause low homogeneity and reproducibility. What is more, the entactin‐mediated gelation of Matrigel is not amenable to chemical modification to regulate mechanical properties, which is not applicable for reprocessing such as bioprinting.^14^ There is an urgency to synthesize alternatives and fabricate new culture matrices with tunable biophysical properties to expand organoid research and application.^15^

Two major solutions exist when it comes to Matrigel alternatives.^16^ Gjorevski et al.^17^ combined a well‐defined 3D matrix based on multiarmed‐polyethylene glycol (PEG) macromers and peptides from fibronectin to fully recapitulate key cues dominating ISC expansion. The synthetic hydrogel with customized contents and tunable mechanical properties (300–1000 Pa) exhibits moderate organoids generation efficiency.^18^, ^19^ Roh et al.^20^ selected natural silk protein to bioengineer epithelial scaffolds. Other natural biomaterials such as hyaluronic acid (HA) and collagen gels also hold potential in organoid culture.^21^, ^22^

Here, we envisaged and fabricated a new bioderived hydrogel with enhanced printability consisting of (i) decellularized extracellular matrix (dECM) pregel from porcine small intestine; (ii) photo‐responsive gelatin methacrylate (GelMA); (iii) photo‐initiator lithium phenyl‐2,4,6‐trimethylbenzoylphosphinate (LAP); and (iv) thickener HA. dECM is a bioactive scaffold from native tissues with cells, functional enzymes and partial biochemical factors removed.^23^ After proper decellularization processes, dECM material consisting of collagens, elastin, fibronectin, and laminin could offer a biomimetic environment with retained native microstructure, cell–ECM interactions, and minor immunogenicity. As a US Food and Drug Administration‐approved medicinal product in use, dECM provides a potential transplantation vector of organoids for regenerative medicine. To enhance the printability of dECM hydrogel, we replenished a biosafe GelMA‐based crosslinked network.^24^, ^25^ GelMA inks are widely utilized in 3D printing for their rapid gelation kinetics and good photo‐curability. Compared to other photopolymers such as PEG diacrylate, poly‐(acrylic acid), or elastic resins, GelMA contains inherent Arg‐Gly‐Asp (RGD) sequences, which are required for organoid encapsulation and proliferation. Also, HA is added to increase the biocompatibility and viscoelasticity and facilitate the cell proliferation and migration.^26^

**SCHEME 1:**
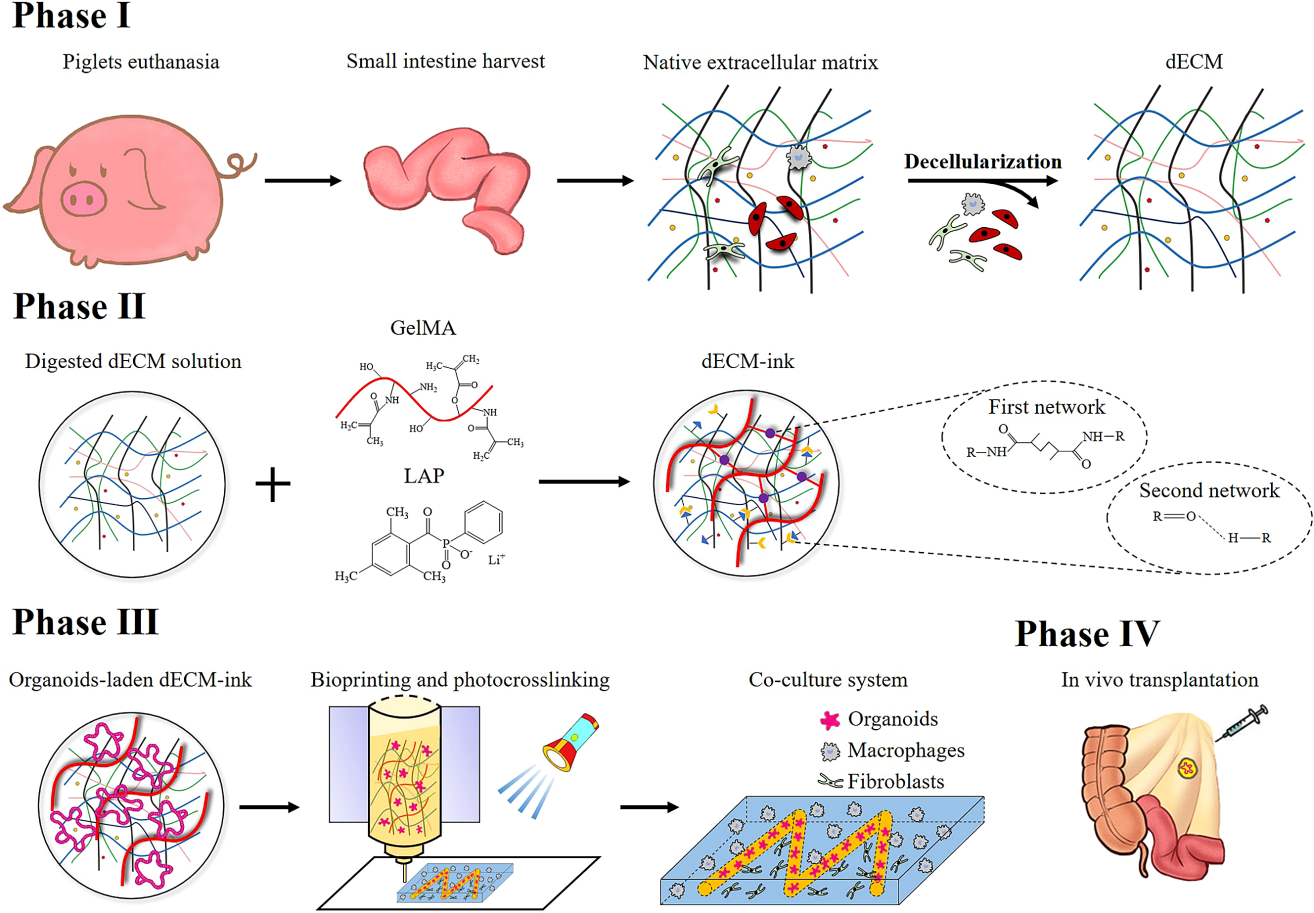
Schematic diagrams of decellularization and fabrication processes of decellularized extracellular matrix (dECM)‐based bioink and blue light‐induced bioprinting method

To verify this hypothesis, we fabricated dECM powder and assessed decellularization effectiveness. Afterward, we prepared dECM‐based bioinks with varied GelMA concentrations and rheological characteristics. We isolated primary small intestinal crypts from C57BL/6 mice and compared in vitro organoid formation in the bioink with that in Matrigel. Differentiation pattern and transcriptomic signature between two groups were analyzed accordingly. A feasible co‐culture system was established via bioprinting of selected bioinks to investigate the crosstalk between printed intestinal organoids and submucosal cells. Furthermore, dECM‐based bioinks containing organoids were transplanted into the mesentery of immune‐deficient NSG mice to verify its applicability for regenerative therapy (Scheme 1).

## 2 RESULTS

### 2.1 Decellularization and characterization of dECM material

As a tissue‐specific material, components of dECM vary according to animal species and gender, tissue origin, and decellularization strategy.^27^ The small intestine tissue from Landrace piglets was used in this study. In order to preserve protein structure under the premise of thorough decellularization, a conjoint processing strategy based on previous published protocols was modified consisting of (i) tissue harvest and rinse to remove mesentery, external layer, and mucosa; (ii) sodium deoxycholate (SDC) and deoxyribonuclease I (DNase‐I) treatment to remove cells and residual DNA; (iii) lyophilization and milling into fine powder; (iv) digestion in pepsin and HCl to gain pregel; and (v) change of pH, salinity, and temperature to initiate gelation (Figure 1a).^24^

A qualitative analysis of decellularized tissue by histological section staining was carried out. Staining of hematoxylin and eosin (H&E) validated the removal of cell components such as nuclei. Staining of Alcian Picrosirius Red (PR) and blue‐periodic acid‐Schiff (AB‐PAS) confirmed the preservation of glycosaminoglycans (GAGs) and multitype collagens (Figure 1b). To meet the standard of dECM, a quantitative analysis of residual DNA in tissue was carried out after SDC and DNase‐I treatment, which showed a significant decrease (Figure 1c). Final DNA content was less than the permitted maximum value 50 ng/mg.^28^ To identify major constituents of dECM powder, a quantitative analysis of collagen, laminin, and elastin was conducted by ELISA. Collagen and elastin showed increased mass proportions compared with those in fresh tissues (Figure 1d). dECM was digested as 6, 8, 10, and 12 mg/ml, whose compressive modulus and turbidimetric gelation kinetics were analyzed. Although the stiffness showed an dECM concentration‐relied increase, the results were close to Matrigel (Figure 1e). At 37°C, changes in absorbance values formed sigmoidal curves, in which the half‐gelation time (*t* 1/2) of 10 mg/ml (5.73 min) and 12 mg/ml (4.68 min) dECM gels were close to Matrigel (5.23 min). While 6 mg/ml (13.53 min) and 8 mg/ml (8.97 min) dECM gels exhibited a delayed gelation process (Figure 1f). Next, we tested the culture efficacy of mouse intestinal organoids within dECM gels. Isolated small intestinal crypts from C57BL/6 mouse were dispersed in the pregels of dECM. Notably, in the first passage, 10 mg/ml dECM gel showed comparable culture efficacy to Matrigel, while the others were inferior to Matrigel (Figure 1g). Among dECM gels, 10 and 12 mg/ml dECM gels could better support the formation of organoids than 6 mg/ml dECM gel.

### 2.2 Fabrication and characterization of dECM‐based bioinks

**FIGURE 2:**
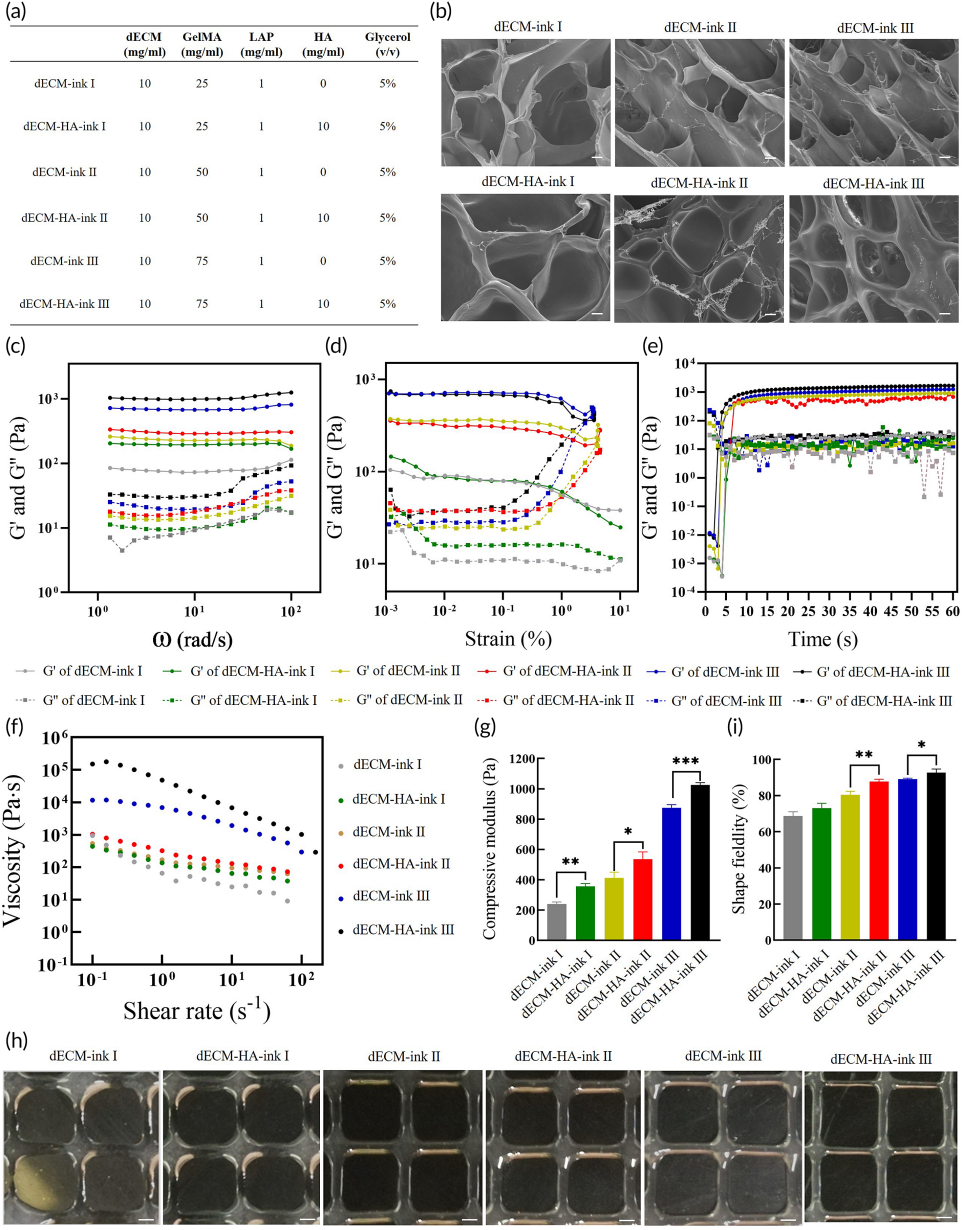
Characterization of fabricated decellularized extracellular matrix‐based bio‐inks. (a) Composite bio‐inks of different concentrations of decellularized extracellular matrix (dECM) powder or gelatin methacrylate (GelMA) for organoids culture and bioprinting. (b) SEM images of dECM‐inks with porous microstructures after gelation. Scale bar 50 μm. (c) Frequency‐sweep of dECM‐inks after gelation. (d) Strain‐sweep of dECM‐inks after gelation. (e) Time‐sweep of dECM‐inks upon blue light irradiation. (f) Viscosity with shear rates of different dECM‐inks. (g) Elastic modulus measured by compressive testing. Mean ± S.D. (*n* = 3 samples). Two‐sided *t*‐test. *p < 0.05, **p < 0.01, and ***p < 0.001. (h) Microscope images of printed lattice patterns with different bio‐inks. Scale bar 200 μm. (i) Measured shape fidelity of printed lattice patterns which is the percentage of the printed pore area relative to the designed value. Mean ± S.D. (*n* = 6 samples). Two‐sided *t*‐test. *p < 0.05 and **p < 0.01

The mechanical properties, shear‐thinning behavior, and viscosity of a bioink are strongly associated with its printability and precision of the construct.^29^ To synthesize an ECM‐based bioink with suitable bioactivity and printability, we chose LAP‐triggered chemical crosslinking between methacrylates, HA, and glycerol to improve the mechanical properties of 10 mg/ml dECM gel, which showed comparable culture efficacy to Matrigel (Figure 2a). We investigated the microstructure of dECM‐inks after gelation. Representative SEM images showed porous microstructures (Figure 2b).^30^ Oscillatory rheological characteristics of dECM‐inks were assessed. dECM‐inks exhibited highly tunable mechanical properties, whose storage modulus (*G*') and loss modulus (*G*") increased with GelMA concentration in frequency‐sweep (Figure 2c). What is more, the adjunction of HA could improve the mechanical strength of dECM‐inks. In time‐sweep, dECM‐inks all underwent a swift gelation (~5 s), which indicated a rapid responsiveness to blue light (25 mW/cm^2^).

In addition, all dECM‐inks exhibited a modest shear‐thinning behavior and HA could increase the viscosity (Figure 2f), which meant that dECM‐HA‐inks were more suitable for extrusion‐based 3D printing. Elastic modulus or stiffness of ECM largely influences or regulates fundamental cellular processes, such as growth, proliferation, migration, differentiation, and organoid formation, making elastic modulus a major index in biomaterial assessment. In this study, elastic modulus of each fabricated bioink was measured through compressive testing (Figure 2g). dECM‐inks exhibited varied but tunable modulus (modulus of dECM‐ink I: 240.23 ± 13.30 Pa; dECM‐HA‐ink I: 355.53 ± 19.21 Pa; dECM‐ink II: 412.74 ± 36.91 Pa; dECM‐HA‐ink II: 536.71 ± 47.70 Pa; dECM‐ink III: 873.65 ± 21.49 Pa; dECM‐ink III: 1027.24 ± 14.74 Pa), which largely depended on the concentration of GelMA and HA.

A 2D patterning test was performed by printing a lattice pattern with 800 × 800 μm^2^ pores, which is one of the most used constructs in tissue engineering. dECM‐ink I and dECM‐HA‐ink I produced unstable constructs (Figure 2h). However, dECM‐HA‐ink II and III showed better printability and shape fidelity compared to their parallels (Figure 2i).

### 2.3 dECM‐based bioinks enable the formation of mouse intestinal organoid

**FIGURE 3:**
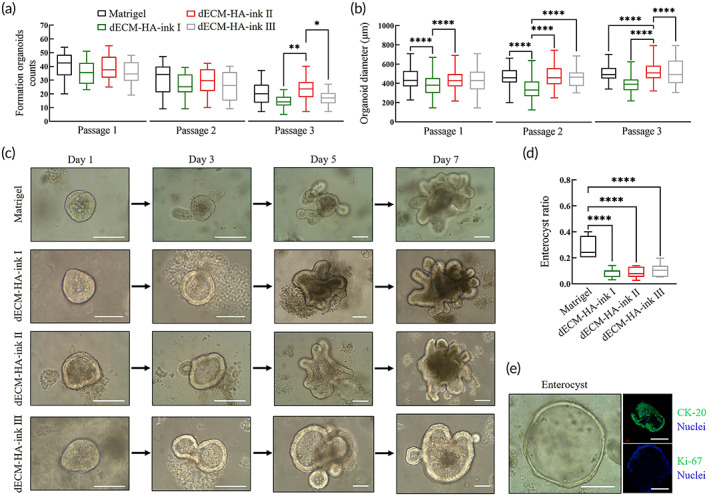
Culture of mouse small intestinal organoids in decellularized extracellular matrix (dECM)‐based bioinks and Matrigel. (a) Formed intestinal organoids per field of view at 100× at Day 7 of three consecutive passages. Mean ± S.D. (*n* = 16 from four organoids cultures). One‐way ANOVA. *p < 0.05 and **p < 0.01. (b) Analysis of organoids diameters at Day 7 of three consecutive passages. Mean ± S.D. (*n* = 80 from four organoids cultures). One‐way ANOVA. ****p < 0.0001. (c) Typical bright field images of formed organoids at Days 1, 3, 5, and 7 at first passage within bioinks and Matrigel. Scale bar 100 μm. (d) Collective ratio of formed enterocysts to cell aggregates at Day 7 of Passage 1. Mean ± S.D. (*n* = 7 organoids cultures). One‐way ANOVA. ****p < 0.0001. (e) Typical bright field image and immunofluorescence analysis of an enterocyst with Matrigel. Showing villi enterocyte marker CK‐20 and proliferation marker Ki‐67. Scale bar 100 μm

We then explored mouse intestinal organoids culture efficacy of fabricated ECM‐based bioinks (Figure 3a). Isolated small intestinal crypts from C57BL/6 mouse were dispersed in pregels of dECM‐HA‐ink I, II, and III, which showed better printability and higher viscosity than their parallels. A canonical factor cluster consisting of R‐spondin, epidermal growth factor (EGF) and Noggin was used as described previously.^7^ ISCs from mouse small intestine showed favorable adaption to dECM‐HA‐inks, which had similar formation organoids counts at Day 7 of two consecutive passages to that of Matrigel. Along with cell passage, the number of formed organoids exhibited a decrease. In Passage 3, dECM‐HA‐ink II showed better culture efficacy compared to dECM‐HA‐ink I and III, indicating a higher proliferation level. For morphological assessment, diameters of organoids at Day 7 of three consecutive passages were analyzed (Figure 3b). In Passage 3, organoids within dECM‐HA‐ink II were characterized by significantly larger size, which also indicated a higher proliferation level.^31^

Budding and expansion of formed organoids at different times of culture were observed by bright field photography (Figure 3c). In all formed organoids, enterocysts accounted for a significantly higher proportion (27.13% ± 8.01%) in Matrigel (Figure 3d). Typical bright field and immunofluorescence images showed the presence of enterocysts, which were rich in enterocyte marker cytokeratin (CK)‐20 and lacking in proliferation marker Ki‐67 (Figure 3e).^32^ Enterocysts demonstrated ISC‐exhausted aggregation of enterocytes with limited life span. Lack of key biochemical stimulations upon ISCs, such as Wnt/R‐spondin signaling in the early stages was considered to cause enterocyst formation.^33^ However, dECM‐HA‐ink I, II, and III exhibited a significantly lower ratio of enterocysts, which indicated that dECM hydrogel could deliver biochemical stimulus and maintain stemness of ISCs more effectively and uniformly compared with Matrigel.^12^

### 2.4 Organoids within dECM‐based bioinks exhibit distinct differentiation pattern

**FIGURE 4:**
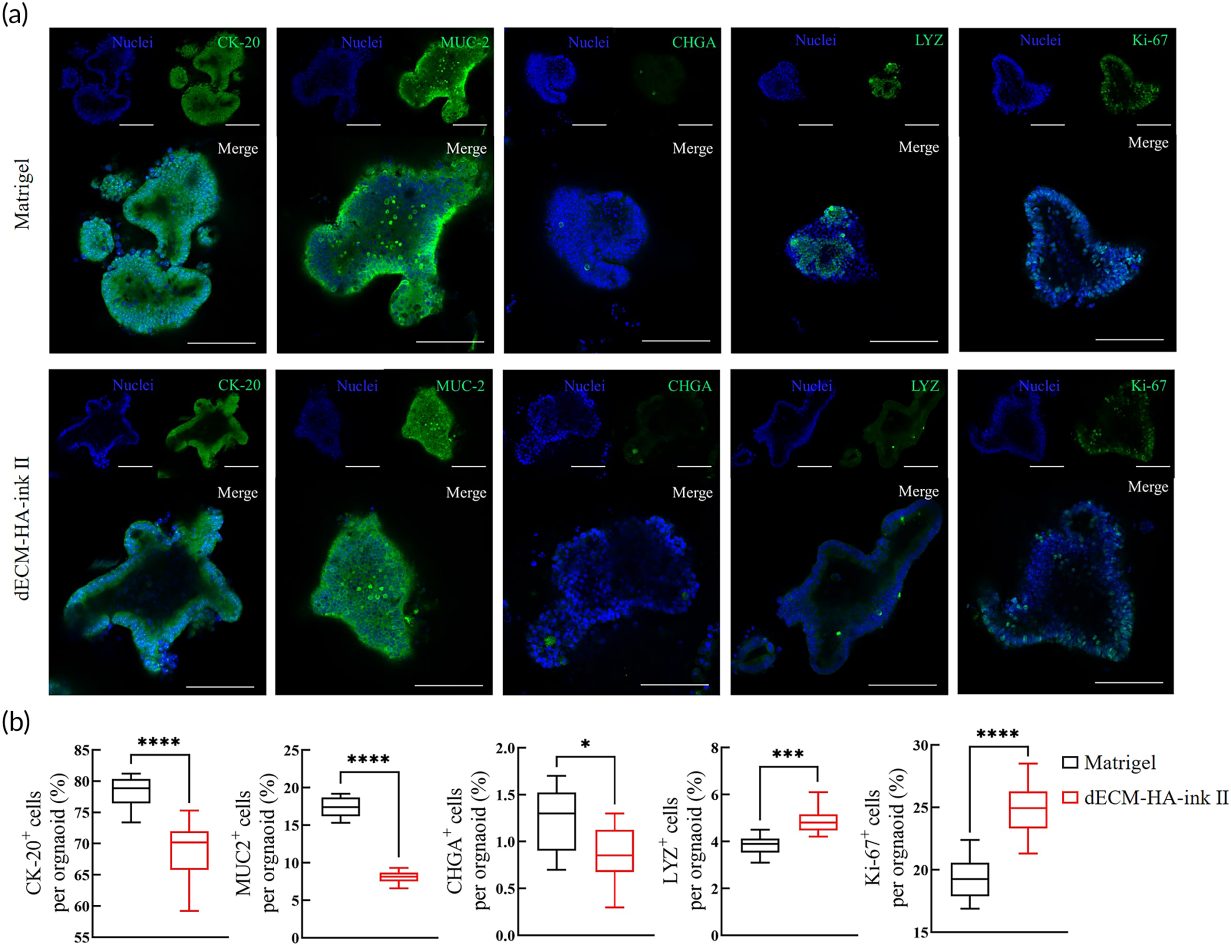
Differentiation pattern of intestinal organoids in decellularized extracellular matrix (dECM‐HA‐ink II) and Matrigel. (a) Whole‐mount immunofluorescence of cultured organoids at Day 7 of Passage 1. Showing villi enterocyte marker CK‐20, goblet cell marker mucin‐2 (MUC‐2), enteroendocrine cell marker chromogranin (CHGA), Paneth cell marker lysozyme (LYZ) and proliferation marker Ki‐67. Scale bar 100 μm. (b) Ratio of differentiated marker positive cells to other cells per organoid. Mean ± S.D. (*n* = 10 organoids). Two‐sided *t*‐test. *p < 0.05, ***p < 0.001, and ****p < 0.0001

To further characterize cultured intestinal organoids and ISC differentiation in dECM‐HA‐ink II, which showed higher culture efficacy than dECM‐HA‐ink I and III, we selected representative intestinal epithelial markers and observed their expression and location by immunofluorescence (Figure 4a). ISCs in dECM‐HA‐ink II were capable of differentiating into enterocytes, goblet cells, enteroendocrine cells, and Paneth cells.

We analyzed the proportions of marker‐positive cells in each organoid to assess differentiation level (Figure 4b). Organoids within dECM‐HA‐ink II exhibited a distinct differentiation pattern compared to those in Matrigel. Organoids within dECM‐HA‐ink II were characterized with significantly higher proportions of LYZ+ Paneth cells (4.87% ± 0.55% compared to 3.82% ± 0.42%) and Ki‐67+ proliferative cells (24.79% ± 2.18% compared to 19.32 ± 1.72%) including ISCs and transit‐amplifying (TA) cells. CK‐20+ enterocytes (68.88% ± 4.67% compared to 78.26% ± 2.50%), mucin (MUC)2+ goblet cells (8.08% ± 0.79% compared to 17.36% ± 1.41%) and chromogranin (CHG)A+ enteroendocrine cells (0.87% ± 0.31% compared to 1.25% ± 0.34%) were found with reduced proportions. The increase in ECM stiffness or other biophysical cues is related to YAP1 activation, which is significant to Paneth cell generation and maturation.^33^, ^34^ As a Wnt source, secretory Paneth cells are vital to maintain ISCs' stemness. Hence, dECM‐HA‐ink II with higher elastic modulus might promote ISC expansion rather than differentiation into short‐lived epithelial cells.

### 2.5 Transcriptomic analysis of organoids within dECM‐based bioink and Matrigel

**FIGURE 5:**
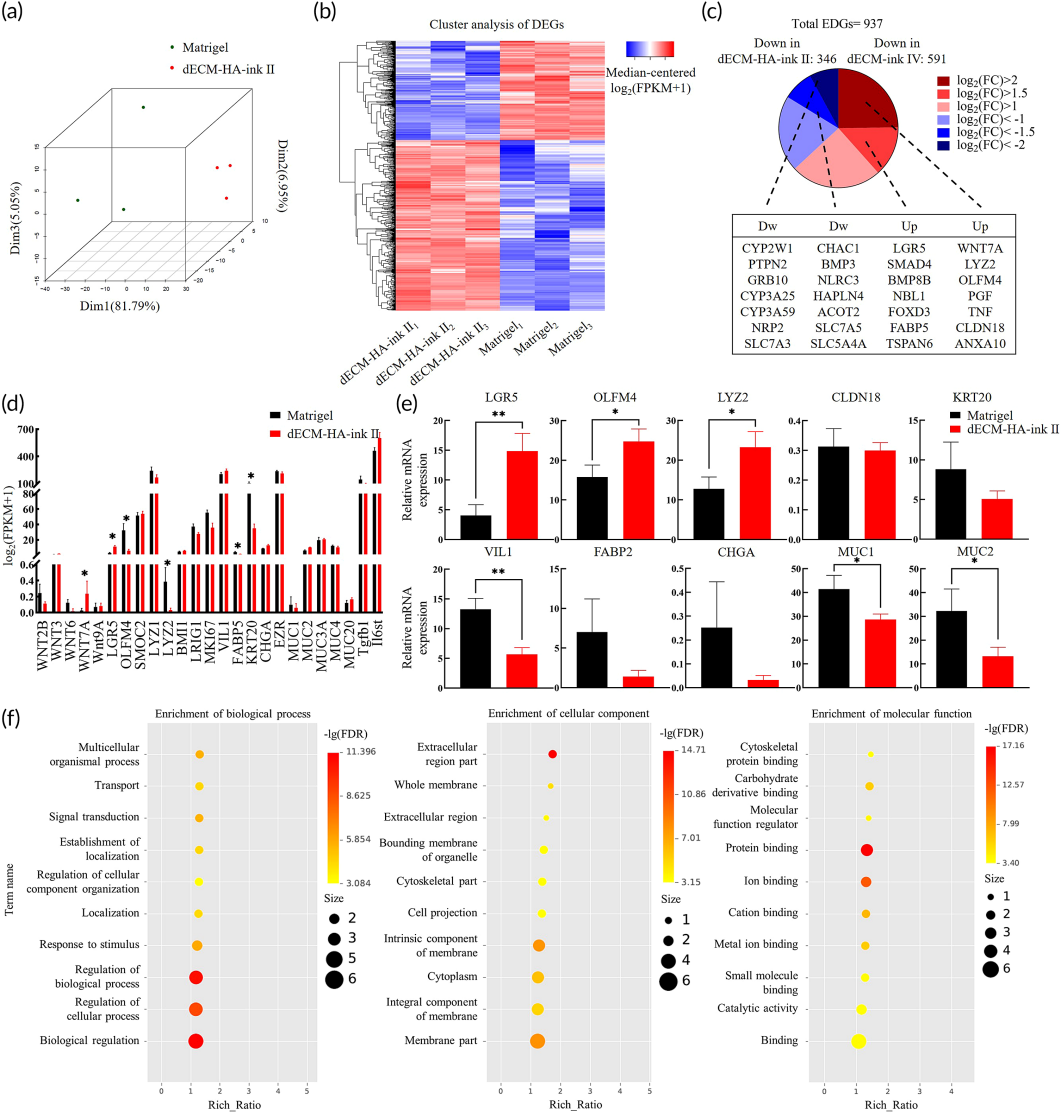
Transcriptomic signature analysis of intestinal organoids in decellularized extracellular matrix (dECM‐HA‐ink II) and Matrigel. (a) 3D principal component analysis (PCA) of dECM‐HA‐ink II and Matrigel group. (b) Heat map of expression of differentially expressed genes (DEGs) ordered according to hierarchical clustering. (c) Pie chart shows numbers of DEGs upregulated and downregulated in dECM‐HA‐ink II compared to Matrigel based on absolute log‐fold change ratios. A selection of DEGs related to intestinal epithelial regulation is shown. (d) Selected genes' expression involved in intestinal stem cells (ISCs) proliferation and differentiation. Mean ± S.D. (*n* = 3 samples). Asterisks indicate DEGs. (e) Relative mRNA expression by quantitative real‐time PCR of selected genes. Gene expression is relative to ACTB gene. Mean ± S.D. (*n* = 3 samples). *p < 0.05 and **p < 0.01. (f) GO categories enriched in DEGs between dECM‐HA‐ink II and Matrigel group

To further reveal the cellular behavior and differentiation feature in dECM‐based bioink, we performed RNA sequencing on formed intestinal organoids (Figure S1a).^31^, ^35^ In principal component analysis (PCA), samples from dECM‐HA‐ink II and Matrigel were clearly separated in Dimension 1 (Figure 5a). Samples from Matrigel exhibited more variability in principal components. Specific transcriptional signatures of samples from dECM‐HA‐ink II and Matrigel were determined by differential expression analysis (Figure 5b). Differentially expressed genes (DEGs) were divided into six distinct clusters according to the K‐means methods ( S1b). High‐expression samples are shown in red and low‐expression samples in blue.

There were 937 DEGs consisting of 591 upregulated and 346 downregulated genes in dECM‐HA‐ink II group (Figure 5c). As shown in the pie chart, canonical ISC markers (e.g., *LGR5* and *OLFM4*) were significantly upregulated in organoids of dECM‐HA‐ink II. Some common crypts markers (e.g., *WNT7A* and *LYZ2*) were upregulated in dECM‐HA‐ink II group, which indicated enhanced ISCs function. Intestinal epithelial markers (e.g., *SLC7A3*, *SLC7A5*, and *SLC5A4A*) and intestinal functional markers (e.g., *CYP2W1*, *CYP3A25*, and *CYP3A59*) were downregulated in dECM‐HA‐ink II, which indicated a defect in epithelial function. However, tight junction proteins (e.g., *CLDN18* and *ANXA10*) were significantly overexpressed in the dECM‐HA‐ink II group, which showed an intact epithelial barrier. We also showed fragment counts of selected genes closely associated with organoid maturation (Figure 5d). TA cell markers (e.g., *BMI1* and *LRIG1*), enteroendocrine cell marker *CHGA*, and goblet cell markers (e.g., *MUC1* and *MUC2*) were comparable with Matrigel. Inflammatory response markers (e.g., *Tgfb1* and *Il6st*) results remained comparable.

A subsequent quantitative polymerase chain reaction (qPCR) analysis was performed to confirm the regulation of transcripts for ISC markers *LGR5* and *OLFM4*, antimicrobial protein *LYZ2*, tight junction protein *CLDN18*, enterocyte markers *KRT20*, *VIL1* and *FABP2*, enteroendocrine cell marker *CHGA*, and goblet cell markers *MUC1* and *MUC2* (Figure 5e). Based on relative mRNA expression, the significant upregulation of ISC markers *LGR5* and *OLFM4* was confirmed in dECM‐HA‐ink II. mRNA expression of Paneth cell marker and antimicrobial protein *LYZ2* also showed an increase. Expression of *CLDN18*, *KRT20*, *FABP2*, and *CHGA* was similar in the two groups. A downregulation of *VIL1*, *MUC1*, and *MUC2* was observed in the dECM‐HA‐ink II group, indicating insufficient differentiation.

To investigate processes relevant in the dECM‐ink role of organoid support, we carried out a functional analysis of Gene Ontology (GO) categories over‐represented in the DEGs (Figure 5f). Transcriptomic analysis highlighted processes consisting of regulation of biological process, biological regulation, extracellular region part and protein binding. Altogether, this difference in differentiation level may arise from variation between native ECM and Matrigel. ISCs formed in dECM were more likely to maintain stemness and build their specific niche environment.

### 2.6 Organoids bioprinting and co‐culture system establishment

**FIGURE 6:**
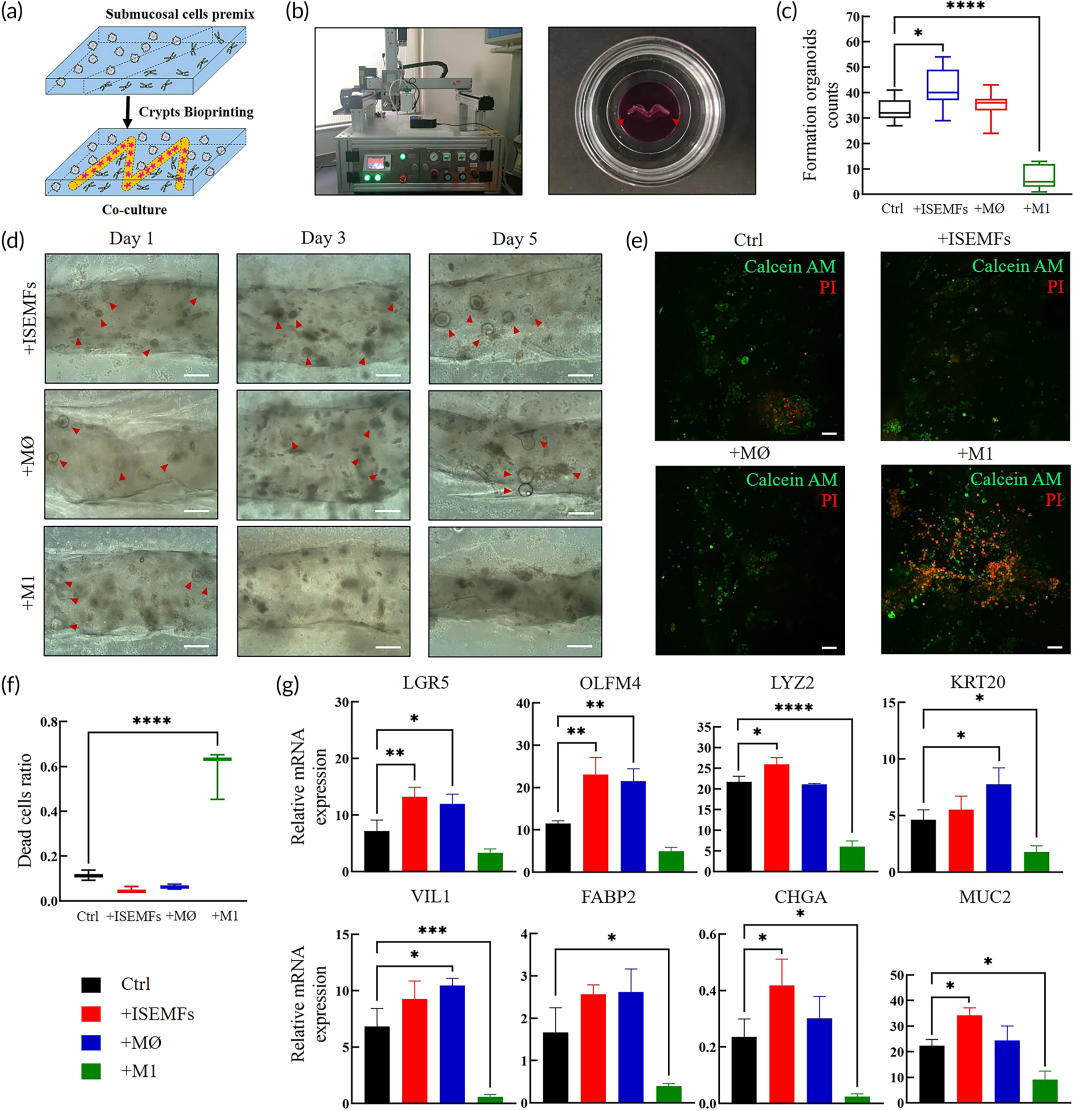
Analysis of fabricated co‐culture system via bioprinting. (a) Bioprinting strategy for organoids co‐culture system establishment. (b) Photograph (left) shows 3D bioprinter combined with blue light source. Photograph (right) shows printed line and circumambient polymerized gelatin methacrylate (GelMA) hydrogel, mixed with phenol red to distinguish from the printed construct. Red arrows indicate printed line. (c) Formed intestinal organoids per field of view at 100× at Day 7 of culture. Mean ± S.D. (*n* = 9). One‐way ANOVA. *p < 0.05 and ****p < 0.0001. (d) Typical bright field images of formed organoids in Days 1, 3, and 5 from different co‐culture groups. Scale bar 50 μm. (e) Live/dead staining of cultured organoids and other cells within hydrogel at Day 7. Scale bar 100 μm. (f) Calculated dead cells ratio of different co‐culture groups according to the immunofluorescence results. Mean ± S.D. (*n* = 9). One‐way ANOVA. ****p < 0.0001. (g) Relative mRNA expression by quantitative real‐time PCR of selected genes. Gene expression is relative to ACTB gene. Mean ± S.D. (*n* = 3 samples). Two‐sided *t*‐test. *p < 0.05, **p < 0.01, ***p < 0.001, and ****p < 0.0001

We attempted to improve conventional culture pattern of organoids using fabricated bioink. To achieve co‐culture of intestinal organoids and submucosal cells in 3D construct, we seeded submucosal cells including ISEMFs and bone‐marrow‐derived macrophages (BMDMs) on pure GelMA/LAP pregel and printed dECM‐HA‐ink II mixed with crypts into the pregel followed by blue light exposure (Figure 6a). We modified our bioprinter with a blue light source and printed 200‐μm wide “M” letter with dECM‐ink (Figure 6b).^36^

Subsequently, we established four culture systems consisting of co‐culture with ISEMFs (+ISEMFs group), co‐culture with nonactivated MØ macrophages (+MØ group), co‐culture with activated M1 macrophages (+M1 group), and intestinal organoids only (Ctrl group). In the +ISEMFs, +MØ, and Ctrl groups, intestinal organoids exhibited robust generation. Formation of organoids at Day 7 in the +ISEMFs group was significantly higher than that in the Ctrl group (Figure 6c). However, organoid formation was significantly decreased in the +M1 group after Day 1 (Figure 6d). To reveal cell expansion level, live/dead staining was performed. In immunofluorescence photographs with printed lines at the center, living cell aggregates were seen in the +ISEMFs, + MØ and Ctrl groups (Figure 6e). However, most cells were dead in the +M1 group, which indicated that epithelial cells were sensitive to inflammatory effects induced by M1 macrophages.^37^ +ISEMFs and + MØ groups were characterized with significantly fewer dead cells (Figure 6f).

To reveal ISC proliferation and differentiation level, qPCR was carried out focusing on representative intestinal markers (Figure 6g). For ISC markers *LGR5* and *OLFM4*, their mRNA expression was upregulated compared to that in the Ctrl group, which confirmed enhanced ISC stemness with ISEMFs and MØ macrophages. It has been reported that subepithelial myofibroblasts and macrophages activate Wnt/R‐spondin signaling as a Wnt source to maintain epithelial homeostasis.^4^ However, differentiation markers such as *KRT20*, *VIL1*, *FABP2*, *CHGA* and *MUC2* were increased in the +MØ group compared to +ISEMFs group, which indicated a differentiation‐promoting effect of residual macrophages in submucosa. What's more, in the +M1 group the expression of ISCs markers (*LGR5* and *OLFM4*) did not exhibit significant decrease compared to the Ctrl group, while an obvious decline in enterocyte marker (*FABP2*), epithelial barrier markers (*VIL1* and *MUC2*), and antibacterial peptide (*LYZ2*) expression was observed, indicating that the terminal‐differentiated enterocytes and epithelial barrier are more vulnerable to the inflammation.

### 2.7 In vivo delivery of the dECM‐ink and Matrigel with intestinal organoids

**FIGURE 7:**
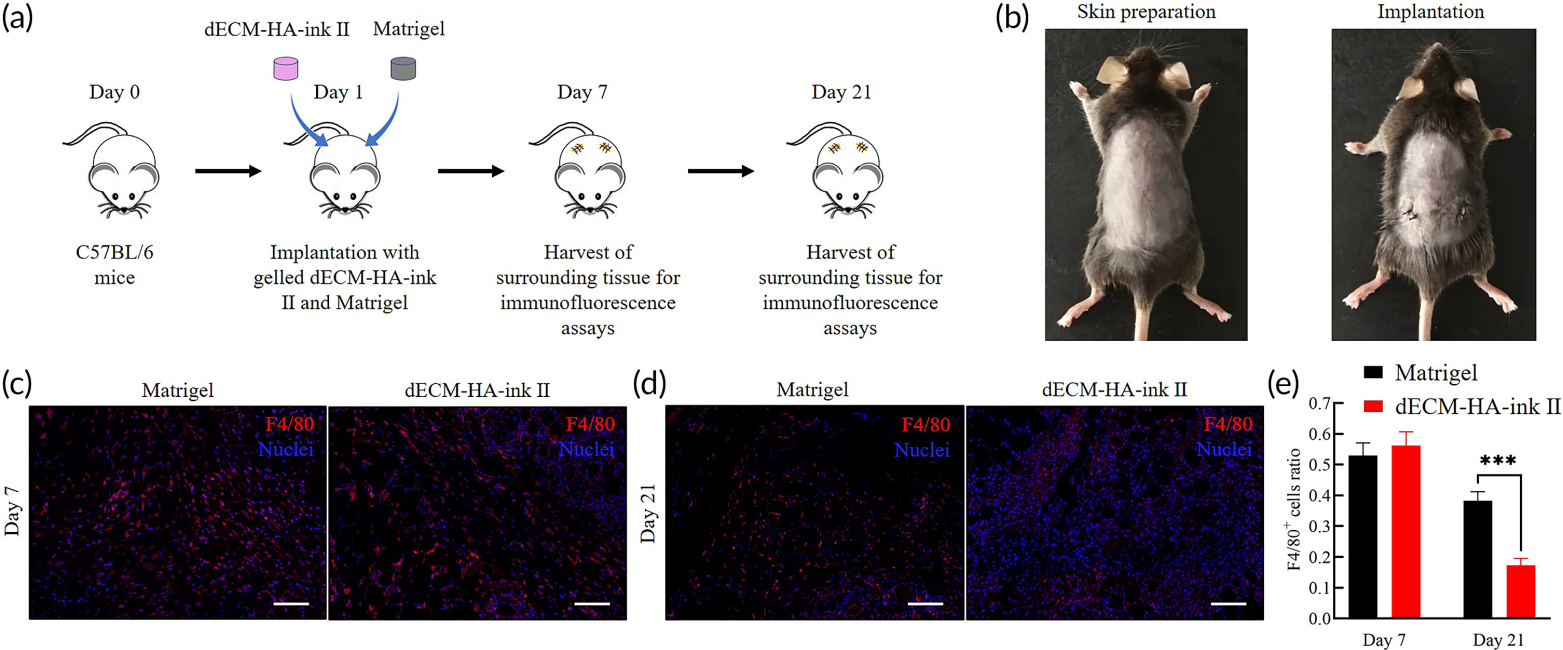
Implantation of decellularized extracellular matrix (dECM‐HA‐ink II) and Matrigel under the back skin of C57BL/6 mice. (a) Schematic diagram shows the implantation process using an animal model of C57BL/6 mice. (b) Photograph (left) shows anesthetized mice after skin preparation. Photograph (right) shows mice with gelled dECM‐HA‐ink II and Matrigel grafted under both sides of the back skin, respectively, and sutured incisions. (c) Whole‐mount immunofluorescence of the tissue around grafts at Day 7. Showing macrophage marker F4/80. Scale bar 100 μm. (d) Whole‐mount immunofluorescence of the tissue around grafts at Day 21. Showing macrophage marker F4/80. Scale bar 100 μm. (e) Collective ratio of F4/80^+^ cells to all cells at Days 7 and 21. Mean ± S.D. (*n* = 3 images). Two‐sided *t*‐test. ***p < 0.001

To evaluate the transplantation potential of fabricated dECM‐based hydrogel, we implanted the gelled dECM‐HA‐ink II and Matrigel under the back skin of 4‐week‐old male C57BL/6 mice to assess tissue's inflammation response to dECM‐HA‐ink II grafts (Figure 7a). Typical immunofluorescence results revealed the gathering of F4/80^+^ macrophages around the grafts at Day 7, which indicated a comparative short‐term inflammatory reaction in both Matrigel and dECM‐HA‐ink II groups (Figure 7c). However, the results at Day 21 exhibited differences.^38^ Immunofluorescence results from the dECM‐HA‐ink II group at Day 21 were characterized with decreased proportion of F4/80^+^ macrophages (Figure 7d,e).

**FIGURE 8:**
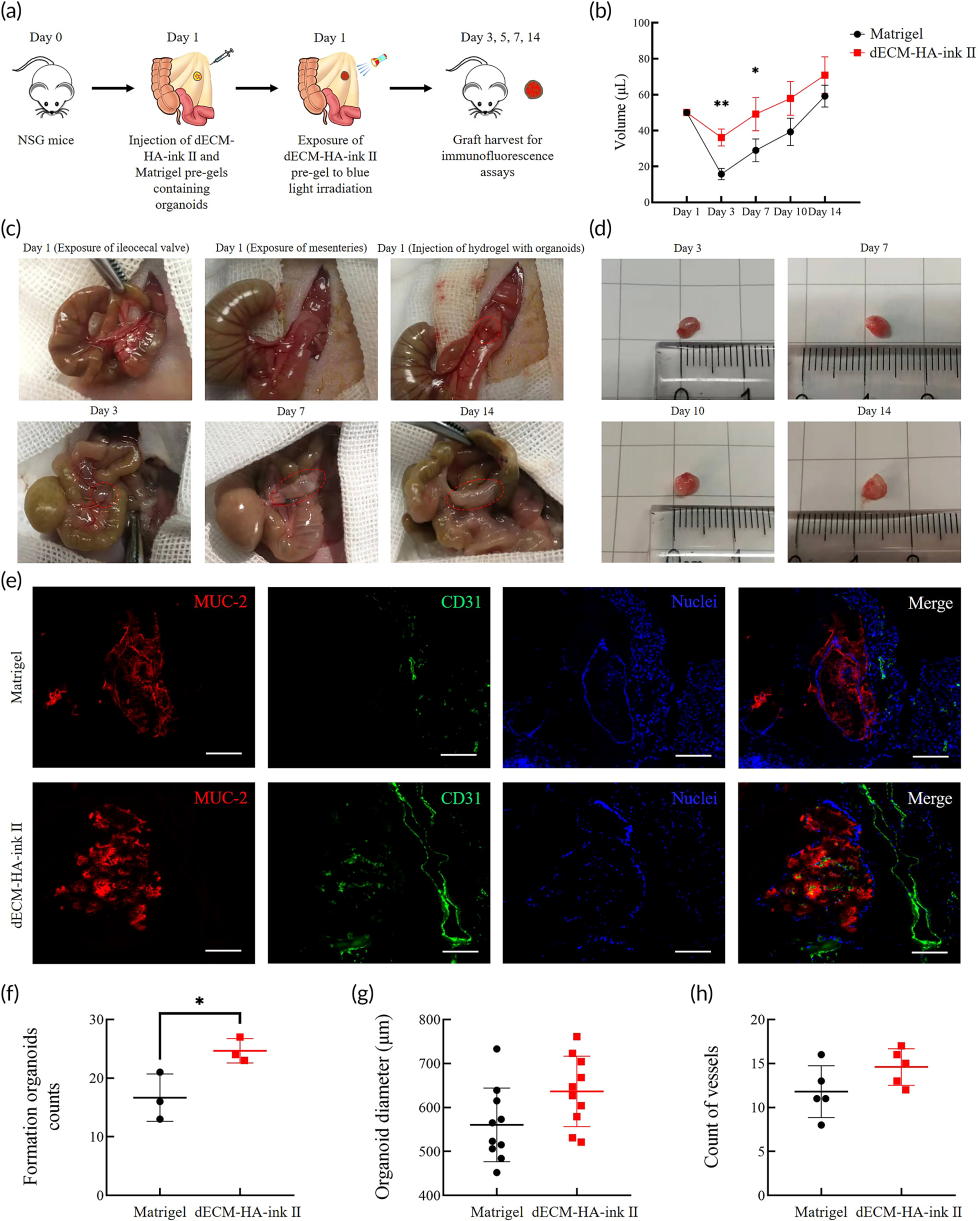
Transplantation of dECM‐HA‐ink II and Matrigel pregels containing organoids into the NSG mice mesentery. (a) Schematic diagram shows the transplantation process using an animal model of NSG mice. (b) Calculated volume of the grafts harvested on Days 3, 7, 10, and 14, respectively. Mean ± S.D. (*n* = 3). Two‐sided *t*‐test. *p < 0.05 and **p < 0.01. (c) Typical images showing the whole procedure of injection and growing graft. (d) Typical images showing harvested grafts on different days after transplantation with increasing size from decellularized extracellular matrix (dECM) group. (e) Frozen sections of matured organoids within the grafts. Showing MUC‐2 and small vessel marker CD31. Scale bar 100 μm. (f) Analysis of the numbers of formed counts on frozen sections from different grafts harvested on Day 14. Mean ± S.D. (*n* = 3 grafts). Two‐sided *t*‐test. *p < 0.05. (g) Analysis of organoids diameters according to the frozen sections of grafts harvested on Day 14. Mean ± S.D. (*n* = 10). (h) Analysis of the numbers of formed small vessels on frozen sections from different grafts harvested on Day 14. Mean ± S.D. (*n* = 5 grafts)

Next, we performed another in vivo delivery experiment with organoids seeded in the pregels of Matrigel and dECM‐HA‐ink II.^39^ We chose 4‐week‐old male NSG mice for organoids transplantation to avoid reject reaction toward allogeneic cells and observe long‐term survival. And we selected mesentery which served as a physiological and anatomic engraftment site (Figure 8a).^39^ After transplantation, the grafts were harvested at Days 3, 5, 7, and 14, respectively, to measure their change in volume, which was relevant to the maturation of organoids within. Notably, dECM grafts suffered a volume loss significantly less than the Matrigel group which might be caused by higher stiffness and different microstructure. After Day 3, grafts from Matrigel and dECM‐HA‐ink II groups all revealed an increasing size (Figure 8c,d). Frozen section was performed on grafts harvested at Day 14 to assess the growth of organoids, which showed unique expression of MUC‐2 and E‐cad proteins (Figure 8e). Furthermore, matured organoids counted from sections of dECM group were significantly outnumbered than that of Matrigel group (Figure 8f), which indicated a better transplantation and growth‐promoting potentials of dECM‐based bioinks. Meanwhile, in order to evaluate the angiogenic potential of dECM‐based bioinks, a CD31 staining was performed to analyze the number of new vessels formed within the grafts, which showed no significant difference (Figure 8h).

## 3 DISCUSSION

Organoid opens an astonishing field with expansive tissue engineering and clinical therapeutic potential. However, lack of matrix material with secured biosafety and flexible tunability is restricting its potential application in regenerative medicine. Poor mechanical properties and printability of Matrigel also limit the development of organoid culture systems and application in bioengineering. Matrigel‐based canonical organoid culture pattern has shown limitations. Here, we describe the development of dECM‐based bioinks with tested biosafety, bioactivity, and tunable mechanical properties, which enable organoid generation and bioprinting.^29^, ^40^ At the same time, as a widely used approach in biomedicine, 3D bioprinting enables the creation of intestinal model or culture scaffolds to provide ISCs with an ink‐based microenvironment to facilitate further application in research and therapeutic medicine.^41^ 3D organoid scaffolds are promising bioengineering tools to construct multicellular systems comprising epithelium, mesenchyme, vasculature, lymph vessels, nerves, and smooth muscles, which may reproducibly direct the fate of ISCs into coordinated and collective behavior.

Subsequent work described culture results of mouse small intestinal organoids within fabricated bioinks. dECM‐based bioinks showed comparable organoid culture efficacy (Figure 3). Interestingly, enterocyst ratio was significantly reduced in bioinks, indicating an altered cell differentiation pattern due to ECM change. Afterward, immunofluorescence photograph and ratio of differentiated marker‐positive cells revealed an increased proportion of proliferative cells and reduced proportion of differentiated cells in organoids of dECM‐HA‐ink II, which may contribute to a significant decline in enterocysts formation (Figure 4). In transcriptomic analysis, several stem cell markers (*WNT7A*, *OLFM4*, and *LGR5*) and tight junction proteins (*CLDN18* and *ANXA10*) were significantly upregulated in organoids of dECM‐HA‐ink II. Expression of intestinal epithelium markers (*SLC7A3*, *SLC7A5*, and *SLC5A4A*) and intestinal function markers (*CYP2W1*, *CYP3A25*, and *CYP3A59*) were downregulated in dECM‐HA‐ink II (Figure 5). Upregulation of extracellular signaling relevant *TSPAN6* was also observed. Combined with qPCR result, organoids within dECM‐HA‐ink II revealed a clear transcriptomic signature different from that of organoids within Matrigel. On the one hand, dECM‐ink with higher stiffness may lead to enhanced activation of mechanical sensors such as YAP1, resulting in increased differentiation toward Paneth cells and enlarged signaling of proliferation. On the other hand, hydrogel that mimics native ECM in content may transmit a collective signal that facilitates the stemness maintaining and proliferation of ISCs which is different from Matrigel.

The homeostasis of the intestinal epithelium in mammals and niche environment of ISCs consist of intestinal flora, epithelium, mesenchyme, vasculature, lymph‐vessels, nerves, smooth muscles, and on. Submucosal fibroblasts, myofibroblasts, macrophages, and endothelial cells are involved in the establishment of biochemical gradients that affect ISC fate and epithelial cell phenotype. However, such crosstalk between stromal components and epithelium is hard to rebuild in canonical organoid systems. By bioprinting, a co‐culture system consisting of intestinal ISCs, primary ISEMFs, BMDMs (MØ and M1) was established (Figure 6).^20^ Organoids co‐cultured with ISEMFs and MØ macrophages showed generation and expansion. Based on qPCR analysis, ISEMFs and MØ macrophages can enhance ISC function and proliferation as a Wnt source. In addition, different submucosal cells exerted different influences on organoid differentiation pattern and epithelial cell phenotype. MØ macrophages may generate a differentiation signal that promotes expression of differentiated cell markers.^42^

So far, finding or synthesizing a vector material for the translational application of organoids remains a huge challenging. The in vivo delivery experiment of dECM gels with cultured organoids in our study makes us realized the potential of combining ECM hydrogels and photo‐responsive crosslink network. The in vivo results reveal that dECM‐based bioink is a suitable vector for organoid transplantation. Nonetheless, 3D printing may bring potential to the pathophysiological studies and functional tissue reconstitution of organoid techniques. We made a successful attempt to combine organoids and bioprinting via fabrication of ECM‐based bioink with suitable bioactivity, biosecurity and printability. These bioinks were characterized with tunable mechanical properties according to GelMA concentration, which enabled us to study the biomechanics behind organoid formation. In addition, via 3D bioprinting, organoid‐laden bioink has been proved capable of fabricating multiform culture systems or scaffolds to facilitate basic research or tissue engineering. By controlling printing parameters and spatial deposition of cells, bioprinted ISCs within the matrix could exhibit spontaneous self‐organization into centimeter‐scale tubular tissues incorporating intestinal features such as continuous lumen, branched vasculature and crypt‐villus domains. Direct bioprinting of biomimetic crypt‐villus structure or sophisticated bioreactors needs to be demonstrated. Bioprinting‐assisted bioengineering may contribute to macroscale organoid tissues that can be applied in regenerative therapy to treat short bowel disease and radiation‐induced intestinal injury.

## 4 CONCLUSION

We fabricated a dECM‐based photo‐responsive bioink with tunable mechanical properties that was able to support ISC proliferation and organoid formation. Organoids within the bioink exhibited enhanced stemness, structural integrity, and compromised differentiation level. Such an organoid differentiation pattern may acquire more physiological features in vivo. Application of native ECM‐mimicking dECM hydrogel might reduce differences in results and increase reproducibility and dependability of organoid research. Bioink with suitable bioactivity, biosecurity and printability enable bioprinting of organoids and fabrication of customized organoid culture systems. A co‐culture model established via bioprinting of organoid‐laden bioink revealed that ISEMFs and MØ macrophages conferred growth‐promoting and variant differentiation‐promoting effects on intestinal organoids. The crosstalk or interaction between epithelial cells and other intestinal components including intestinal flora and mesenchyme may be further elaborated by this feasible co‐culture system. Lastly, according to our findings from in vivo delivery experiment, dECM‐based bioink may expand the translational application of organoids of human origin.

## 5 METHODS

### 5.1 Preparation of porcine small intestinal submucosal tissue

Fresh whole small intestine of male Landrace (Sus scrofa domesticus) piglets up to 3 kg was purchased from Jiangsu Hurun Agricultural Products Co. Ltd. Decellularization of the whole small intestine submucosa was performed according to reported protocols with some modifications.^43^ The mesentery and external layer of the small intestine were removed. The internal layer consisting of mucosa and submucosa was cut off longitudinally and fully rinsed and cleaned with pressurized water. Later, a scalpel handle was used to scrape off fluffy mucosa. Submucosal tissue was cut into 5‐cm pieces.

### 5.2 Fabrication of dECM from porcine small intestinal submucosa

Each batch of dECM was fabricated from three piglets' submucosal tissue. A conjoint decellularization strategy modified from established protocols for porcine intestine was used subsequently. To initiate decellularization, submucosal tissue pieces were placed in pure water (Spring‐R10; RSJ, Xiamen, China) overnight at 4°C. Washed tissue was decellularized in 4% SDC (30970; Sigma‐Aldrich, St. louis, MO, USA) for 4 h at room temperature. After SDC treatment, the tissue was washed with pure water for 24 h at 4°C, with water change every 6 h. Afterwards, a step of 2000kU DNase‐I (11284932001; Sigma‐Aldrich) in 1 M NaCl was performed for 3 h at room temperature. The tissue was washed with pure water again for 48 h at 4°C, with water changed every 6 h. A rotator at 60 rpm (DS‐S 100; Servicebio, Wuhan, China) was used throughout the decellularization and rinsing. Rinsed tissue was lyophilized (LGJ‐12; HUAYU XIONGDI, Zhengzhou, China) for 72 h and milled (Tissuelyser‐24; Jingxin, Shanghai. China) into a fine powder that could pass through a 70 μm cell strainer (BS‐70‐XBS; Biosharp, Anhui, China). Acquired dECM powder was stored at −20°C until further use.

### 5.3 Digestion and gelation protocol of dECM

For cell culture, stored dECM powder underwent a sterilization step of 70% (v/v) ethanol for 2 h at 4°C and two rinses with pure water.^27^ dECM powder was digested in pepsin/HCl solution (P6887; Sigma‐Aldrich) (1 mg/ml in 0.01 M HCl) at 6/8/10 mg/ml for 72 h at room temperature under constant magnetic stirring (PC‐420D; Corning, Corning, NY, USA) to obtain pregel. Large particles were discarded via a step of centrifugation at 2000 rpm for 5 min (Megafuge 8R; Thermo Fisher, Waltham, MA, USA). Pregel was transferred to cold storage at 4°C to avoid unexpected gelation. The 0.1 M NaOH solution was used to neutralize pregel to physiological pH of 7.4. The pregel was equilibrated to cytocompatible salinity by adding 10% 10× PBS for mechanical analysis or 10× DMEM/F12 (PM150312P; Procell, Wuhan, China) for cell culture. After thoroughly mixing, the dECM pregel underwent gelation at 37°C for 30 min.

### 5.4 Tissue histology

Samples were taken randomly from decellularized submucosa to initiate paraffin embedding. Samples were fixed in 4% paraformaldehyde solution in PBS for 1 h at room temperature, dehydrated, paraffin embedded, and cut into 5‐μm sections. H&E staining was performed on tissue slides to confirm the absence of nuclei. AB‐PAS and PR were used to assess the presence of GAGs and collagen, respectively.

### 5.5 Quantification analysis of DNA

Samples were taken at random after harvesting, after SDC treatment and after DNase‐I treatment. DNA content was assessed by a PureLink Genomic DNA Mini Kit (K182000; Thermo Fisher). Final DNA concentration was measured by a NanoDrop microvolume spectrophotometer (ND‐ONEC‐W; Thermo Fisher). Samples from three different batches were tested.

### 5.6 Quantification analysis of dECM

Samples were taken from pregel after digestion and neutralization to quantify major dECM contents by Porcine Collagen ELISA Assay kits (J03851; Jining, Shanghai, China), Porcine Elastin ELISA Assay kits (N06616; Jining) and Porcine Laminin ELISA Assay kits (J03685; Jining). Final contents concentration was measured by a spectrophotometer (PT‐3502PC; Potenov, Beijing, China).

### 5.7 Turbidity

Pregel samples from dECM‐gel and Matrigel (356231; Corning) was taken to measure turbidity using a spectrophotometer (PT‐3502PC; Potenov). Two hundred microliters of pregels were pipetted into a 96‐well plate. Absorbance at 450 nm was measured at 37°C once per min for 1 h. Measured results were standardized to a PBS control group to calculate normalized absorbance (NA) using the formulation below. R is the absorbance reading measured at a selected time. R_min_ is the smallest absorbance reading. R_max_ is the highest absorbance reading.

$$\mathrm{NA}=\frac{R-R_{\min}}{R_{\max}-R_{\min}}$$

### 5.8 Fabrication of dECM bioinks

Neutralized dECM pregel was stored at 4°C to avoid unexpected gelation. To fabricate composite hydrogel, 25/50/75 mg/ml fractional GelMA (M299512; Aladdin, Shanghai, China), 1 mg/ml LAP (L157759; Aladdin) and 10 mg/ml HA (H131007; Aladdin) were added to dECM pregel. Constant magnetic stirring for 1 h at 30°C was needed to dissolve completely. Well‐distributed mixed gel was supplemented with 5% v/v glycerol to enhance printability. Newly fabricated dECM bioink can be stored at 4°C for 1 week. Before usage, stored dECM bioink needed transient heating at 30°C for 10 min to depolymerize crosslinks between gelatin chains driven by low temperature.

### 5.9 SEM analysis

Hydrogel samples were prepared (4 mm in diameter and 10 mm in height). The samples were frozen at −80°C and then freeze‐dried during 48 h. The specimens were cracked, sputter‐coated with Pt and examined using SEM (S‐3400N; Hitachi, Tokyo, Japan).

### 5.10 Mechanical properties analysis

Rheological properties of dECM‐inks after gelation were studies using a rheometer (MCR302; Anton Paar, Graz, Austria). The constant frequency was fixed at 10 Hz in the oscillatory strain sweep experiment; the constant strain was fixed at 1% in the oscillatory frequency sweep experiment at 25°C. The angular frequency (*ω*) was swept from 0.1 to 100 rad/s. The viscosity of pregels was measured with shear rates ranging from 0.01 to 1000/s at 25°C. Elastic modulus of hydrogels was measured by compressive testing. Cylindrical samples (5 mm in diameter and 8 mm in height) were installed on an instron machine (CMT4202; Jiehu Co. Ltd. Chengdu, China) and compressed slowly at a rate of 1 mm/min. Compressive modulus was obtained according to the plotted stress–strain curve based on recorded compression distance and corresponding force at 10% strain.

### 5.11 Printability test and organoids bioprinting

The bioprinting system consisted of an *XYZ*‐axis stage, mechanical dispenser, pneumatic pressure‐assisted dispenser (DLH200; Donglihu, Nanjing, China) and blue light source (LS1601; Engineering for Life, Suzhou, China).^41^ dECM‐inks were loaded into a 1 ml syringe connecting a 200‐μm nozzle. The printability test was performed at a dispensing rate of 0.5735 μl/s. dECM‐inks were extruded at a printing speed of 10 mm/min. Printed lines were recorded using a microscope (XD‐202; Jiangnan, Nanjing, China). For organoid printing, isolated mouse intestinal crypts were loaded into pregels at a density of 5 × 10^5^/ml.

### 5.12 Culture of mouse intestinal organoids

Small intestinal crypts were isolated and cultured from 4‐week‐old male C57BL/6 mice.^7^ Ethics approval was obtained by the Animal Ethics Committee of Jingling Hospital (2021DZGKJDWLS‐00106). After euthanasia via cervical dislocation, intestine from the cecum to the stomach was harvested and rinsed with multiple change of 4°C PBS. The inner lumen was cut off longitudinally and softly scraped with a throat swab to remove remaining mucus and contamination. The tissue was cut into small pieces (1 mm wide) and transferred into a 50‐ml falcon tube containing 20 ml 5 mM EDTA‐PBS. The tube was incubated with consistent rotation at 4°C for 40 min. Vigorous shaking by hand was used to facilitate rinsing and isolation processes at intervals. After incubation, the supernatant was filtered through a 70‐μm cell strainer. Filtered cell solution underwent centrifugation at 100 × *g* at 4°C for 5 min. Acquired cell plates were resuspended with DMEM/F12 (KGM12500; KeyGEN BioTECH, Nanjing, China) and columnar crypts were calculated using a hemocytometer (075‐03‐001; ISOLAB, Eschau, Germany). Cultured crypts were encapsulated at a density of 1500 per 50 μl hydrogel (Matrigel: DMEM/F12 media = 4: 1) or 5 × 10^5^ per 1 ml bioink. Complete crypt media consisting of advanced DMEM/F12 containing 15% fetal bovine serum (G11‐70500; Genial Biological, Suite A, Brighton, Colorado, USA), 1% penicillin/streptomycin (KGM0023; KeyGEN BioTECH), 50 ng/ml EGF (AF‐100‐15‐100; PeproTech, Rocky Hill, New Jersey, USA), 100 ng/ml Noggin (250–38; PeproTech) and 250 ng/ml R‐spondin (315–32; PeproTech). Medium was changed every 3–4 days. Organoids were passaged every 6–8 days by manual disruption. Cell recovery solution (354253; Corning) at 4°C was used to dissolve Matrigel.

### 5.13 Quantification of formed organoids, organoid diameter, and enterocyte ratio

In fields of view at 100× (*n* = 16), formed organoids were counted.^31^ Fields close to the dome center and border were avoided. Major diameters of formed organoids at Day 7 of each passage (*n* = 80) were analyzed. Among organoids, spheroid aggregates characterized with larger lumen, thinner cell layer and absence of columnar cells were regarded as enterocysts. Enterocyst ratio was calculated according to enterocyst and organoid counts in fields of view at 100× (*n* = 7).

### 5.14 Immunostaining

A batch of three domes (50 μl) of cultured organoids was dissolved by manual disruption to initiate immunostaining.^44^ After centrifugation (100 × *g*, 4°C, 5 min), sedimentary organoids were fixed in 4% paraformaldehyde solution in PBS for 30–60 min. To block and permeabilize organoids, 1 ml Triton X‐100 and 2 g BSA were added to 1 L PBS to prepare organoid wash buffer (OWB). The fixed organoids were permeabilized using OWB for 20 min. Primary antibodies (anti‐Ki‐67, 11‐5698‐82; anti‐E‐cadherin, 53‐3249‐82; Thermo Fisher; anti‐CK‐20, ab109111; anti‐MUC2, ab272692; anti‐lysozyme, ab108508; anti‐CHGA, ab254322; Abcam, Cambridge, UK) were incubated in OWB (1:100 dilution) at 4°C overnight in rotation (60 rpm).^45^ After extensive washing, second antibodies (GB22301, GB22303; Servicebio) were incubated in OWB (1:200 dilution) at 4°C overnight on rotation (60 rpm). Nuclei (KGA215; KeyGEN BioTECH) and live/dead (KGAF001; KeyGEN BioTECH) staining was performed. Prepared organoids were transported into a glass bottom dish and observed using confocal microscopy (LSM900; Zeiss, Jena, Germany).

### 5.15 RNA sequencing

Cultured organoids were dissolved by manual disruption and rinsed to remove matrix that could interfere with RNA harvest. RNA was harvested using Trizol reagent (KGA1201; KeyGEN BioTECH) formyl trichloride and isopropanol.^35^ The high quality and concentration (≥10 nM) of RNA samples was confirmed using 2100 RNA Nano 6000 Assay Kit (Agilent Technologies, Santa Clara, CA, USA). Enriched mRNA was fragmented and subjected to reverse transcription. The amplified cDNA was sequenced using Illumina HiSeq2500 (Illumina, San Diego, CA, USA) with a read length of 100 bp. A heat map of DEGs (*p* ≤ 0.05 and |log2(FC)| > 1) was analyzed via hierarchical cluster method based on K‐means clustering. The y axis depicted the results of hierarchical clustering.

### 5.16 Real‐time qPCR

After total RNA harvest, cDNA was prepared using Superscript IV cDNA synthesis kit (Thermo Fisher).^32^ Real‐time PCR was performed using a QuantStudio 6 Flex (Applied Biosystems, Foster City, CA, USA). Primer sequences are listed below.

**Table:** 

Primer name	Forward	Reverse
VILLIN 1	ATGACTCCAGCTGCCTTCTCT	GCTCTGGGTTAGAGCTGTAAG
LGR5	ACCCGCCAGTCTCCTACATC	GCATCTAGGCGCAGGGATTG
LYZ2	GGAATGGATGGCTACCGTGG	GGAATGGATGGCTACCGTGG
CHGA	CTCGTCCACTCTTTCCGCAC	CTGGGTTTGGACAGCGAGTC
MUC2	ATGCCCACCTCCTCAAAGAC	GTAGTTTCCGTTGGAACAGTGAA
OLFM4	CAGCCACTTTCCAATTTCACTG	GCTGGACATACTCCTTCACCTTA
KRT20	TTCAGTCGTCAAAGTTTTCACCG	TCCTATACAGCGAGCCACTCA
FABP2	GTGGAAAGTAGACCGGAACGA	CCATCCTGTGTGATTGTCAGTT
MUC1	GGCATTCGGGCTCCTTTCTT	TGGAGTGGTAGTCGATGCTAAG
CLDN18	ACATGCTGGTGACTAACTTCTG	AAATGTGTACCTGGTCTGAACAG

### 5.17 Culture of ISEMFs and macrophages

ISEMFs were isolated based on published protocols.^5^ After euthanasia via cervical dislocation, rinsed small intestinal tissue from 7 day‐old male C57BL/6 mice was cut into 0.5‐mm^2^ pieces and transferred into a T25 flask. Dispase at 0.31 mg/ml (17105041; Gibco, Grand Island, NY, USA) was used to digest rinsed tissue. After incubation at room temperature for 30 min on rotation, the supernatant underwent multistep centrifugation to acquire pellets containing ISEMFs. Medium for ISEMFs consisted of DMEM high‐glucose (KGA12800; KeyGEN BioTECH), 15% fetal bovine serum, 1% penicillin/streptomycin and EGF 50 ng/ml.

To acquire BMDMs, BM was extracted from the tibia and femur of 4‐week‐old male C57BL/6 mice following removal of surrounding muscle.^46^ The bone marrow was flushed into a Petri dish using a 1‐ml syringe filled with PBS. Cell suspension was centrifuged (250 × *g*) at room temperature for 5 min to pellet cells. Harvested BMDMs were cultured in macrophage medium consisting of DMEM high‐glucose, 10% fetal bovine serum, 1% penicillin/streptomycin and macrophage colony‐stimulating factor (M‐CSF) (96‐315‐02‐10; MultiSciences Biotech, Hangzhou, China) 50 ng/ml for 7 days with medium changes every other day to obtain MØ macrophages. For M1 polarization, MØ macrophages were cultured in macrophage medium supplemented with 100 ng/ml lipopolysaccharide (LPS) (KGR0048; KeyGEN BioTECH) and 10 ng/ml murine interferon (IFN)γ (96‐315‐05‐20; MultiSciences Biotech) for 1 day before co‐culture. For storage, BMDMs were frozen in 10% dimethyl sulfoxide and 90% macrophage medium.

### 5.18 Establishment of co‐culture system

Prepared ISEMFs, MØ and M1 macrophages were seeded into dECM‐free pregel consisting of 50 mg/ml GelMA and 1 mg/ml LAP at a density of 1 × 10^6^/ml, respectively.^20^ The submucosal cell‐laden pregel was transferred into a glass bottom dish. The crypt‐laden dECM‐HA‐ink II was extruded in the layer of dECM‐free pregel. After printing, blue light was used to initiate gelation of two hydrogels within the dish. No submucosal cells were added to the Ctrl group. Organoid culture medium consisting of DMEM/F12, 15% fetal bovine serum, 1% penicillin/streptomycin, 50 ng/ml EGF, 100 ng/ml Noggin and 250 ng/ml R‐spondin was used in the +ISEMFs and Ctrl groups. For the +MØ group and + M1 groups, we used organoid culture medium supplemented with 50 ng/ml M‐CSF.

### 5.19 In vivo implantation

Four‐week‐old male C57BL/6 mice (*n* = 6) were anesthetized using 4% chloral hydrate via intraperitoneal injection. Two incisions (0.5 cm) were performed on the back of the mice. A dECM‐HA‐ink II of 200 μl was gelled into a shape of cylinder under blue light irradiation and implanted under the right back skin. Then a Matrigel of 200 μl was implanted under the left back skin after gelation in an incubator at 37°C for 15 min. Afterward, the incisions were sutured. At Day 7, three mice were sacrificed via cervical dislocation and the tissue around the grafts was fixed in 4% paraformaldehyde solution for immunostaining analysis (anti‐F4/80, Q61549, Servicebio). The other three mice were sacrificed at Day 21 following same protocols.

### 5.20 In vivo transplantation

Four‐week‐old male NSG mice (*n* = 24) were anesthetized using 4% chloral hydrate via intraperitoneal injection. An abdominal incision (1.2 cm) was performed to find ileocecal valve and expose nearby mesenteries. For dECM‐HA‐ink II group (*n* = 12), 4000 matured organoids which had been cultured for 3 days were seeded within 50 μl pregel of dECM‐HA‐ink II. Then the 50 μl dECM‐HA‐ink II pregel containing organoids was injected into the thickened part of the mesentery. The injection was followed by 10 s long exposure to blue light irradiation. For Matrigel group (*n* = 12), 4000 matured organoids, which had been cultured for 3 days were seeded within 50 μl pregel of Matrigel. And the mixture was injected into the thickened part of the mesentery. For both dECM‐HA‐ink II and Matrigel group, at Days 3, 5, 7 and 14 after transplantation, three mice were sacrificed via cervical dislocation for graft size measurement. Grafts harvested at Day 14 were quick‐frozen in liquid nitrogen followed by immunostaining analysis. For organoids maturation demonstration, formed organoids on frozen sections from different grafts were counted (*n* = 3). Organoid diameters were also collected from different sections (*n* = 10).

### 5.21 Statistical analysis

Differences between the experimental groups were analyzed using Two‐sided *t*‐test or one‐way ANOVA. RNA sequencing relevant PCA analyze, pie plot and hierarchical clustering were performed on ANNOROAD (v. 2018). Genomic data were filtered using Microsoft Excel (v. 2016). Other graphs were performed on GraphPad Prism (v. 8.0).

## AUTHOR CONTRIBUTIONS

**Zi‐Yan Xu:** Conceptualization (equal); data curation (lead); formal analysis (lead); methodology (lead); software (lead); validation (lead); writing – original draft (lead); writing – review and editing (lead). **Jin‐Jian Huang:** Conceptualization (equal); investigation (equal); project administration (equal); supervision (equal). **Ye Liu:** Investigation (equal); methodology (equal). **Can‐Wen Chen:** Investigation (equal); methodology (equal). **Gui‐Wen Qu:** Investigation (equal); methodology (equal). **Ge‐Fei Wang:** Funding acquisition (equal); project administration (equal); supervision (equal). **Yun Zhao:** Conceptualization (equal); formal analysis (equal); funding acquisition (equal). **Xiu‐Wen Wu:** Conceptualization (equal); supervision (equal); writing – review and editing (equal).

### PEER REVIEW

The peer review history for this article is available at https://publons.com/publon/10.1002/btm2.10327.

## Supporting information


**Supplementary Fig. 1 Volcano plot and K‐means clustering. a** Volcano plot shows changes in expression between dECM‐HA‐ink II and Matrigel group. Selected gene are colored in red. **b** K‐means clustering analysis of DEGs. Red line indicates gene clusters with FPKM equal to 1.

**Supplementary Fig. 2 Immunofluorescence of newborn small vessels. a** Whole‐mount immunofluorescence of the grafts from Matrigel group. Showing small vessel marker CD31. Scale bar 100 μm. **b** Whole‐mount immunofluorescence of the grafts from dECM‐HA‐ink II group. Showing small vessel marker CD31. Scale bar 100 μm.

## Data Availability

The data that support the findings of this study are available from the corresponding author upon reasonable request.
