# Supplementary figures and images for: Extracellular matrix bioink boosts stemness and facilitates transplantation of intestinal organoids as a biosafe Matrigel alternative
Source: Bioeng Transl Med. 2022 Apr 26;8(1):e10327. doi: 10.1002/btm2.10327 (PMC9842023; doi:10.1002/btm2.10327)

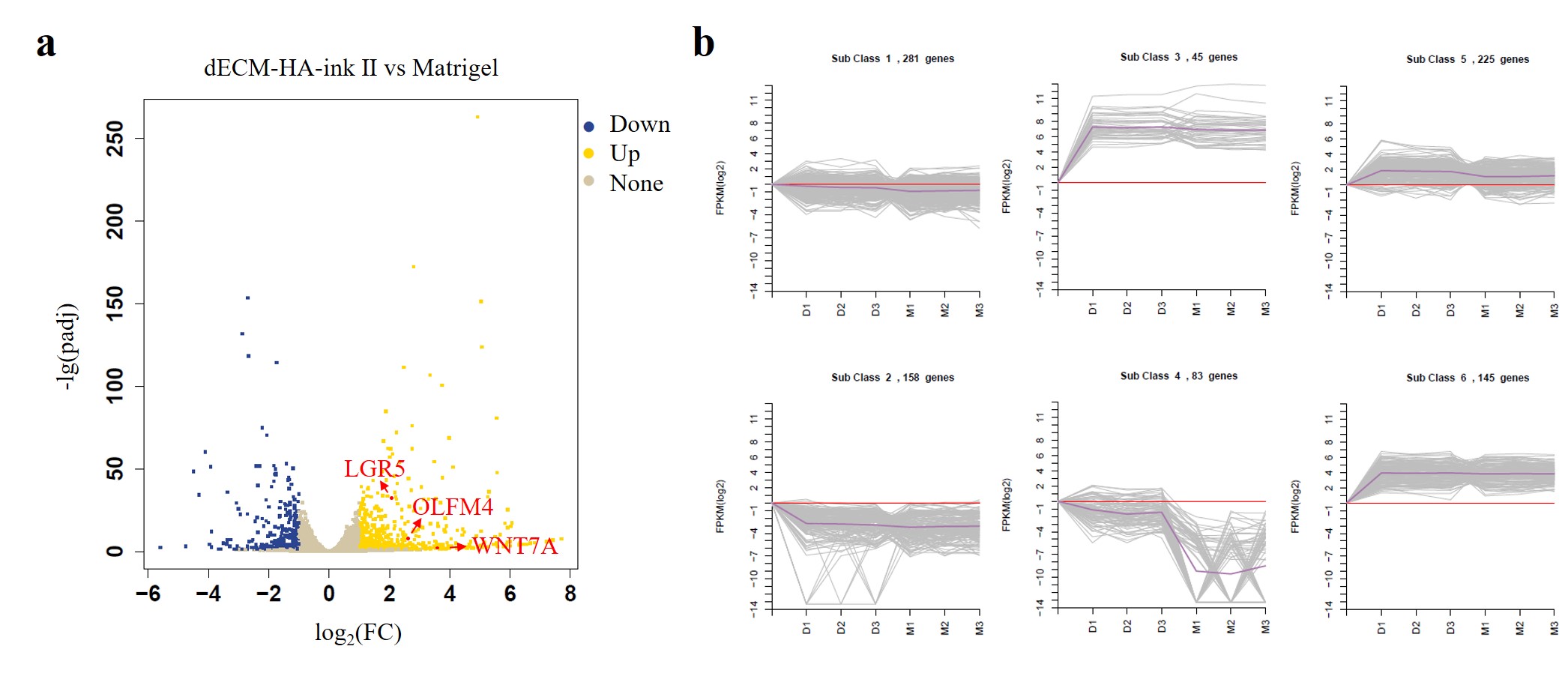

Supplement: Supplementary file 1 — Supplementary Fig. 1 Volcano plot and K‐means clustering. a Volcano plot shows changes in expression between dECM‐HA‐ink II and Matrigel group. Selected gene are colored in red. b K‐means clustering analysis of DEGs. Red line indicates gene clusters with FPKM equal to 1. [file BTM2-8-e10327-s001.jpg]

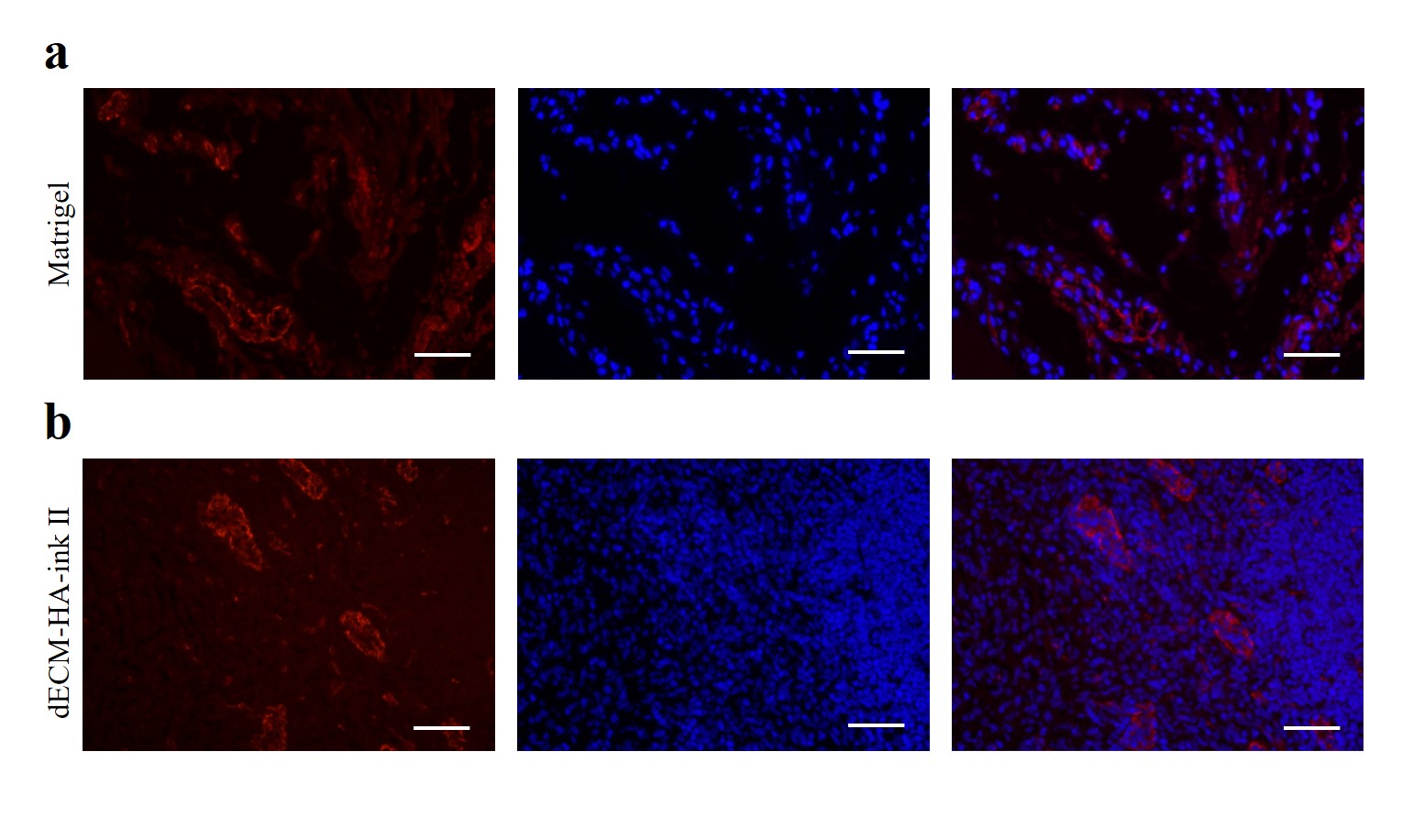

Supplement: Supplementary file 2 — Supplementary Fig. 2 Immunofluorescence of newborn small vessels. a Whole‐mount immunofluorescence of the grafts from Matrigel group. Showing small vessel marker CD31. Scale bar 100 μm. b Whole‐mount immunofluorescence of the grafts from dECM‐HA‐ink II group. Showing small vessel marker CD31. Scale bar 100 μm. [file BTM2-8-e10327-s002.jpg]
